# Noise-induced transitions of the Atlantic Meridional Overturning Circulation in CMIP5 models

**DOI:** 10.1038/s41598-020-76930-5

**Published:** 2020-11-18

**Authors:** Daniele Castellana, Henk A. Dijkstra

**Affiliations:** 1 Department of Physics, Institute for Marine and Atmospheric Research Utrecht, Utrecht University, Utrecht, The Netherlands; 2 Department of Physics, Centre for Complex Systems Studies, Utrecht University, Utrecht, The Netherlands

**Keywords:** Climate sciences, Ocean sciences

## Abstract

By studying transition probabilities of the Atlantic Meridional Overturning Circulation (AMOC) in an ensemble of CMIP5 climate models, we revisit one of the stability indicators of the AMOC, i.e. the freshwater transport carried by the AMOC at the southern boundary of the Atlantic basin. A correction to this indicator, based on the transition probabilities, is suggested to measure whether an AMOC state is in a multiple equilibrium regime or not. As a consequence, the AMOC of all CMIP5 models considered is in a multiple equilibrium regime and hence, in principle, a collapsed AMOC state should exist in each of these models. The results further demonstrate the dependence of the Atlantic surface freshwater flux on the AMOC and the impact of extreme events in the AMOC on temperatures in the North Atlantic region.

## Introduction

The Atlantic Meridional Overturning Circulation (AMOC) plays a crucial role in the climate system, as it transports about 1.5 PW of heat northward in the Atlantic Ocean. Observations from the RAPID project have shown evidence of a decrease of a few Sverdrups (Sv) in the strength of the AMOC over the period 2004–2017^1^. It is well known^2–4^ that the AMOC strength is sensitive to the surface freshwater forcing. In particular, model results^5–8^ indicate that the AMOC can have more than one stable state under the same surface forcing conditions. The physics behind the multistability of the AMOC is well understood and due to the salt-advection feedback^2^.

Projections from state-of-the-art climate models from the IPCC (Intergovernmental Panel on Climate Change, 2013) indicate that the AMOC will weaken under the effect of the anthropogenic climate change at a rate up to about 1 Sv per decade^9^. When studying the output of climate models which are part of the CMIP5 (Coupled Model Intercomparison Project Phase 5)^10^, the stability of the AMOC cannot be determined easily because of the long simulation time required^11^. This is one of the reasons why multiple equilibria have not been found in such models. Another reason might be that the AMOC states are not in a multiple equilibrium regime, possibly due to biases in the models^12^. Anyway, it cannot be excluded that the AMOC in the present-day climate is in a multiple equilibrium regime^13^.

To determine whether a multiple equilibrium regime exists in a model, indicators of the AMOC stability have been developed. The quantity $M_{ov}$^14^ is defined as the amount of freshwater transported by the AMOC at the southern boundary of the Atlantic Ocean (at $34^\circ \hbox {S}$). A negative (positive) value of $M_{ov}$ indicates that the AMOC is in a multiple (single) equilibrium regime. $M_{ov}$ has proven to be a good indicator for the detection of a multiple equilibrium regime in ocean-only models^15,16^, and it has been used to interpret the behaviour of the AMOC in several coupled models and in observations^14,17–21^. However, it was only developed in the framework of ocean-only models, as it ignores feedbacks between ocean and atmosphere^11,12^.

When the present-day AMOC is in a multiple equilibrium regime, changes in the freshwater forcing may induce a transition to a weak or ‘collapsed’ AMOC state. When such a transition is not induced by the crossing of a critical boundary, but by fast variability in the forcing (‘noise’), it is called a noise-induced transition^22^. In a recent study^23^, such noise-induced transitions were studied in a box model of the Atlantic Ocean, where $M_{ov}$ is a perfect indicator for the multiple equilibrium regime of the AMOC. It was found that two types of noise-induced transitions can occur: a full collapse of the AMOC, referred to as S-type transition, and a partial and temporary collapse, associated with a temporary cessation of the downwelling in the North Atlantic, referred to as F-type transition. While an S-type transition is unlikely to occur within the next 100 years, the likelihood of an F-type transition strongly depends on the amplitude of the freshwater noise and the value of $M_{ov}$^23^.

It is important to investigate the noise-induced transitions of the AMOC in CMIP5 models as these transitions can have substantial consequences for the climate of the North Atlantic. Several studies have shown the impact of the AMOC variability on the sea surface temperature (SST) of the North Atlantic Ocean^24,25^, as well as its influence on the European climate^26,27^. *Roberts et al.*^28^ computed regression maps of changes in SST, associated with a change in the AMOC, using decadal mean time series from an ensemble of CMIP5 models, and found positive correlations in the North Atlantic. The area that seems to be the most sensitive to the AMOC variability is the Atlantic Subpolar Gyre. *Rahmstorf et al.*^29^ developed an AMOC index, based on the SST difference between the Atlantic Subpolar Gyre and the Northern Hemisphere, which was found to correlate well with the AMOC strength. *Duchez et al.*^30^ investigated the link between the observed AMOC anomalies at $26^\circ \hbox {N}$ and satellite based Atlantic SST anomalies and showed that there is a significant correlation between these quantities.

The main aim of this paper is to analyse noise-induced AMOC transitions in CMIP5 model results. *Castellana et al.*^23^ already indicated that F-type transitions can be found in CMIP5 models and preliminary results were shown in their Supplementary Information^23^. In “Results” section, we detect F-type transitions in CMIP5 models and connect these to $M_{ov}$. Moreover, the consequences of noise-induced transitions in the AMOC strength are explored, especially with regard to anomalies in the surface temperatures of the North Atlantic region. A summary and discussion of the results follow in “Summary and discussion” section. “Models and methods” section provides an overview of the model output and the methodology used.

## Results

**Figure 1:**
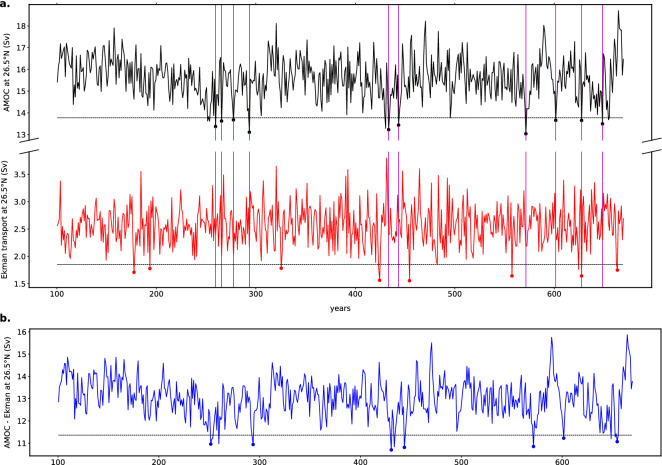
(**a**) Yearly-averaged time series of $\Psi _M$, i.e. the maximum value of the AMOC at $26.5^\circ \hbox {N}$ (black) and of $\Psi _E$, i.e. the zonally integrated Ekman transport (red), respectively, in the pre-industrial control simulation of the model MIROC5. The dashed horizontal lines indicate, for each time series, the lower bound of the $2 \sigma$ confidence interval around the mean. The disks identify the time points at which extreme events occur, defined as the values extending below the threshold. Note that some of the values are not marked, because of the (10-year) clustering. The magenta vertical lines are drawn in correspondence with the extreme events of $\Psi _M$ on top of both the time series. In this way, it is possible to qualitatively check whether a certain extreme event in the AMOC is related with anomalies in the wind-stress variations. (**b**) The difference time series $\Psi _M^{cor} = \Psi _M - \Psi _E$.

In Fig. 1a, the time series of the AMOC strength $\Psi _M$ and the Ekman transport $\Psi _E$ at $26^\circ \hbox {N}$ are shown for the MIROC5 model. Before the time series were annually averaged, they were detrended by removing the first EOF (Empirical Orthogonal Function) obtained with the Singular-Spectrum Analysis (SSA)^31^. SSA has proven to be an effective way to make sure that trends are removed from the time series: the stationarity is a necessary requirement in order to investigate extreme events. It is clear that several of the extreme events occurring in the AMOC cannot be attributed to extremes in the Ekman transport. Therefore, such events are interpreted as being buoyancy-induced and to be able to isolate these transitions we use (as explained in detail in “Models and methods” section) the time series $\Psi _M^{cor} = \Psi _M - \Psi _E$ (Fig. 1b).

Extreme events (or transitions) like the ones highlighted in Fig. 1b, extending below the $2 \sigma$ threshold, can be found in all the CMIP5 models investigated here. Using the procedure described in “Models and methods” section, we are able to compute, for each model, the transition probability of such events; the values are shown in Table 1. At first glance, it is surprising that, although the models show very similar values of noise and transition probability, their values of $M_{ov}$ are quite different. In particular, four of the models present a positive $M_{ov}$, which would indicate that the AMOC is in a single equilibrium regime, hence unable to undergo transitions^23^.

**Table 1:** Table containing the list of the models used in this study.

# model	Model name	Institute ID	$\Psi _M$	$M_{ov}^{34^\circ S}$ (Sv)	$\eta$	*P*	$M_{ov}^{cor}$ (Sv)
1	ACCESS1-0	CSIRO-BOM	14.7	$-$ 0.038	0.27	0.63	$-$ 0.14
2	ACCESS1-3	CSIRO-BOM	16.5	$-$ 0.004	0.30	0.70	$-$ 0.13
3	CNRM-CM5-2	CNRM-CERFACS	15.7	$+$ 0.039	0.11	0.81	
4	GFDL-CM3	NOAA GFDL	20.6	$+$ 0.104	0.20	0.55	$-$ 0.16
5	GFDL-ESM2M	NOAA GFDL	22.6	$+$ 0.150	0.22	0.80	$-$ 0.18
6	MIROC5	MIROC	15.5	$-$ 0.036	0.20	0.81	$-$ 0.19
7	MPI-ESM-LR	MPI-M	18.9	$+$ 0.002	0.19	0.69	$-$ 0.19
8	MPI-ESM-MR	MPI-M	16.6	$-$ 0.117	0.16	0.75	$-$ 0.21
9	MPI-ESM-P	MPI-M	18.3	$-$ 0.014	0.19	0.66	$-$ 0.18
10	MRI-CGCM3	MRI	14.4	$-$ 0.006	0.12	0.80	$-$ 0.23

Each model is identified with a certain number, which is used in the following figures to refer to it. The other columns of the table represent, respectively, the AMOC strength, the value of $M_{ov}$ obtained by *Mecking et al.* ^17^, the noise and the transition probability calculated with the procedures described in “Models and methods” section, and the corrected values of $M_{ov}^{cor}$.

**Figure 2:**
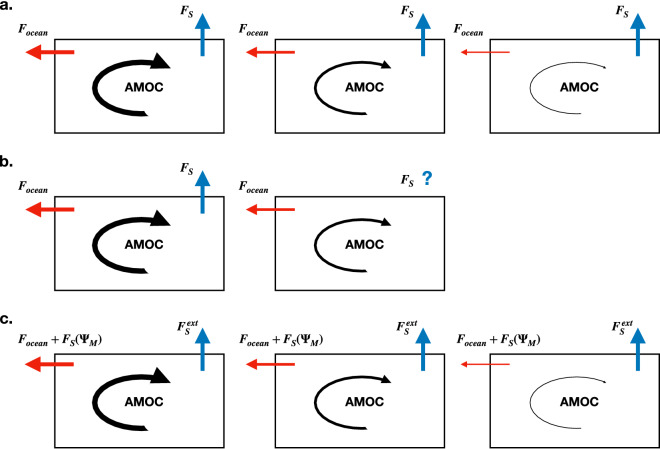
(**a**) Illustration of the positive feedback mechanism established with negative $M_{ov}$, in a ocean-only model. In the first figure, the AMOC is in equilibrium and it transports freshwater ($F_{ocean}$) outside of the Atlantic ocean; $F_S$ represents the constant net freshwater flux between ocean and atmosphere. In the second figure, the AMOC weakens due to a perturbation: therefore, less freshwater is transported outside of the basin ($F_{ocean}$ decreases). As a consequence, the basin becomes fresher and the AMOC further weakens, as depicted in the third figure. (**b**) Same situation, for a coupled model: the freshwater flux is no longer constant, therefore a weakening of the AMOC results in a change in its magnitude. Hence, no conclusions can be drawn about a potential feedback mechanism. (**c**) Same situation, for a coupled model, with $M_{ov}^{cor} < 0$. The component of the atmospheric freshwater flux dependent on the AMOC strength $F(\Psi _M)$ is included in the definition of $M_{ov}$ and $F_S^{ext}$ is constant. Hence, the original idea behind $M_{ov}$ is retrieved.

While there could be several other reasons for this result, we interpret it here as an indication that $M_{ov}$ is not an adequate indicator of the multiple equilibrium regime in climate models. To explain our idea for a correction, we recall the physics behind $M_{ov}$ (see Fig. 2a for an illustrative scheme). When $M_{ov}$ is negative, the overturning circulation transports freshwater out of the Atlantic basin ($F_{ocean}$ in the figure): if a perturbation is introduced in the system, such that the AMOC weakens, less freshwater is transported out of the basin; therefore, the Atlantic Ocean becomes fresher, which, in turn, further weakens the AMOC. When $M_{ov}$ is positive, a weakening of the AMOC results in a saltier basin, which has the effect of stabilising the circulation. In other words, a positive (negative) feedback mechanism on the AMOC strength is established when $M_{ov}$ is negative (positive). However, the mechanism explained above is valid only provided that atmospheric fluxes are not affected by the AMOC. This assumption is certainly valid for ocean-only models, where fixed surface fluxes are prescribed. On the other hand, when coupled models are taken into consideration, the atmospheric feedbacks cannot be ignored^32^: a weakened AMOC results in a variation in the surface freshwater flux over the Atlantic ocean (Fig. 2b). An improved indicator for the AMOC stability needs to take such feedbacks into consideration^11^.

Corrections to $M_{ov}$ due to salinity biases in CMIP5 models^33^ have already been suggested^17^, but do not really take into account the effect of the AMOC on the freshwater flux. In the following, we take a bold step to develop a correction of $M_{ov}$ by making use of the relation between transition probabilities, freshwater noise and $M_{ov}$ as determined in the box model of *Castellana et al.*^23^, where $M_{ov}$ was a perfect indicator of the multiple equilibrium regime. It is known that box models can quite accurately represent AMOC transitions^34^. Although this approach is quite a leap, fortunately there is an interesting check at the end on the consistency of the procedure.

**Figure 3:**
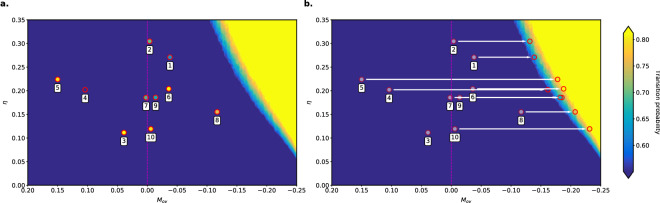
(**a**) Transition probabilities of the AMOC transitions in 100 years, adapted from *Castellana et al.*^23^, as function of $M_{ov}$ and atmospheric noise $\eta$. The dashed magenta line indicates $M_{ov} = 0$, which is supposed to separate the monostable from the bistable regions. On the top of the contour map, circles were drawn in correspondence of the $M_{ov}$ values and the noise amplitudes calculated from the models (5th and 6th columns of Table 1, respectively). The transition probabilities calculated from the models, indicated by the colors of the coloured circles, do not match with the ones predicted by the box model. (**b**) The circles in (**a**) were shifted horizontally, by correcting the values of $M_{ov}$ with the procedure described in the text. The values (based on the box model) of the transition probability were calculated for each value of the parameters ($M_{ov}$, $\eta$) and the $M_{ov}$ of each CMIP5 model has been corrected, such that the calculated transition probability fits the one in the box model.

As expected, the probability of F-type transitions in *Castellana et al.*^23^ was found to be higher with lower (more negative) values of $M_{ov}$ and with higher noise amplitudes. Based on these results, we calculate the value of $M_{ov}$ that each CMIP5 model should have, based on the values of noise and transition probabilities calculated. This is done by inverting the map in Fig. 4 of *Castellana et al.*^23^ and interpolating $M_{ov}$ on a grid constructed with the other two variables (the atmospheric noise $\eta$ and the transition probability *P*). The corrected values of $M_{ov}$ are shown in the last column of Table 1. A graphical representation of the method is shown in Fig. 3. Note that the correction for the model CNRM-CM5-2 is missing: this is due to the fact that the corrected value of $M_{ov}$ should be less than $-\, 0.25$ Sv; unfortunately, the actual value cannot be obtained with the method previously described, as in the box model, transition probabilities were not calculated for such large negative values of $M_{ov}$.

The discrepancy between the original values of $M_{ov}$ and their corrected values is here interpreted as being due to the presence of atmospheric feedbacks on the AMOC. Consider the Atlantic freshwater budget of a General Circulation Model (GCM). In equilibrium, this budget relates the integrated freshwater flux over the Atlantic basin, including the Arctic Ocean, ($F_S$) with the freshwater transports by the AMOC and the horizontal gyre circulation through^14^:

1 $$\begin{aligned} F_S = M_{ov} + M_{az} \end{aligned}$$

where $M_{az}$ represents the freshwater transport by the southern Subpolar Gyre circulation. In equation (1), we have neglected small-scale mixing contributions and Bering Strait transport as these terms are usually much smaller than the others^12^. Several studies^35^ have shown that the net evaporation over the Atlantic basin depends on the strength of the AMOC, i.e., a lower evaporation is associated with a weaker AMOC. This motivates to split the term $F_S$ into two contributions:

2 $$\begin{aligned} F_S = F_S(\Psi _M) + F_S^{ext} \end{aligned}$$

where the first term in the right hand side represents the dependence on the AMOC strength $\Psi _M$, whereas the second term is the net evaporation over the Atlantic Ocean in the absence of an AMOC.

A corrected value of $M_{ov}^{cor}$ should take into account the effect of the variation of the AMOC on the transport of freshwater inside/outside the basin, hence

3 $$\begin{aligned} M_{ov}^{cor} + M_{az} = F_S^{ext} \end{aligned}$$

Therefore, using the relations (1) and (2), we obtain

4 $$\begin{aligned} M_{ov}^{cor} = M_{ov} - F_S(\Psi _M) \end{aligned}$$

An illustration of the mechanism is given in Fig. 2c: when $M_{ov}^{cor}$ is negative, it cannot be stated whether the AMOC transports freshwater outside or inside the Atlantic basin (the direction of $F_{ocean}$ is unknown). However, if the component of the atmospheric freshwater flux dependent on the AMOC strength $F(\Psi _M)$ is included in the definition of $M_{ov}$, we can imagine as if a net freshwater transport was flowing outside of the basin, partly via the southern border of the Atlantic ocean, and partly through the atmosphere ($F_{ocean}$ + $F(\Psi _M)$). When the AMOC weakens due to a perturbation, this net transport decreases, while $F_S^{ext}$ remains constant, by definition. Therefore, the Atlantic ocean becomes fresher, which, in turn, makes the overturning circulation weaker. One could in principle extrapolate the dependence of $F_S$ on the AMOC strength $\Psi _M$ using additional CMIP5 model simulations and calculate the corresponding value of $M_{ov}$ to determine $M_{ov}^{cor}$. Unfortunately, such results are not available for CMIP5 models, and in fact an enormous computational effort would be required to compute all these equilibrium solutions.

**Figure 4:**
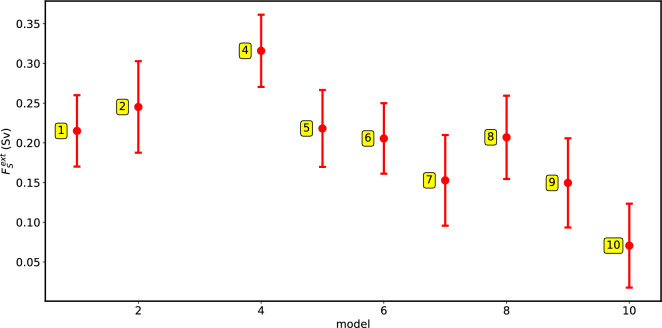
$F_S^{ext}$, computed for each CMIP5 model with the procedure explained in the text. The error bars represent the confidence interval of one standard deviation around the mean values, calculated for the original time series $F_S$.

However, and here comes an internal check of the procedure, it is expected that the different CMIP5 models should have about the same $F_S^{ext}$. If transition probabilities were not connected to the correct stability indicator, here proposed to be $M_{ov}^{cor}$, then there would be an enormous spread in $F_S^{ext}$ values. From the CMIP5 models, we can evaluate $F_S^{ext}$ from (combining (2) and (4))

5 $$\begin{aligned} F_S^{ext} = F_S + \Delta M_{ov} \end{aligned}$$

where $\Delta M_{ov} = M_{ov}^{cor} - M_{ov}$ and $F_S$ is just the surface freshwater flux calculated from the control simulation. The results (Fig. 4) are quite astonishing as the mean value of $F_S^{ext}$, averaged over the 10 models, is 0.20 Sv, with a standard deviation of only 0.06 Sv! This result is highly nontrivial as the correction $M_{ov}^{cor}$ is only based on the transition probabilities. We see this as an indication of an internal consistency of the, admittedly bold, assumptions made.

In order to investigate the consequences of the occurrence of AMOC extremes for the surface temperatures of both land and ocean areas in the North Atlantic region, we selected three areas: Western Europe, Greenland and the Atlantic Subpolar Gyre (Fig. S1 in Supplementary Section A). The time series obtained by integrating the surface temperature field over the three subregions were yearly-averaged and detrended. Surface temperatures differ from SSTs only when sea ice is present but when the data is yearly-averaged, the differences in temperature anomalies are very small (not shown). The time series for the temperature anomalies in the three regions, once again for the model MIROC5, are shown in Fig. S2 in Supplementary Section A, together with the $\Psi _M^{cor}$ time series. Several of the temperature anomalies seem to be correlated with extremes in the AMOC. In order to give a statistical measure of such correlation, for each subregion, we calculate a synchronisation index between the extreme events in the AMOC and those in the surface temperature, using the Event Synchronisation (ES) algorithm^36^. This method is preferred over more traditional correlation methods, since our focus is on the extreme events, and not on the whole time series. Moreover, the advantage of ES is that it allows to study interrelations between series of non-Gaussian data or data with heavy tails^37,38^. The algorithm, described in detail in Supplementary Section B, is applied to two time series (indicated as *i* and *j*, respectively) and it allows to calculate two quantities: $Q^{ij}$ and $q^{ij}$. Here, $Q^{ij}$ represents the strength of the synchronisation, with $Q^{ij} = 1$ indicating full synchronisation (e.g., between a time series with itself), and $Q^{ij} = 0$ an absence of synchronisation. The quantity $q^{ij}$ determines the lag between the time series, with $q^{ij} > 0$ ($< 0$) indicating that the events in the time series *j* precede (follow) the events in the time series *i*.

**Figure 5:**
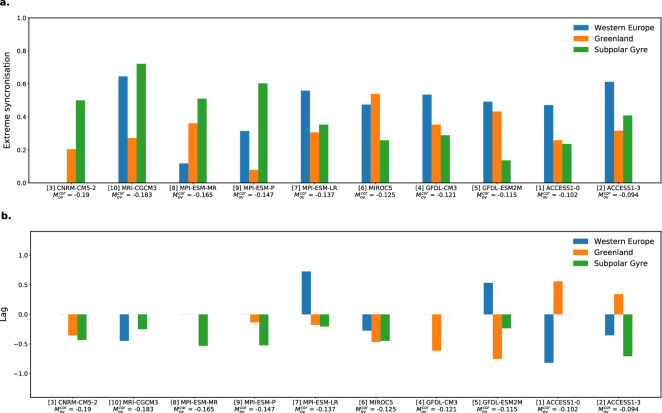
(**a**) Event synchronisation strength *Q*, calculated, for each CMIP5 model, between the $\Psi _M^{cor}$ anomalies and the surface temperature anomalies obtained for three different subregions in the North Atlantic (Fig. S1). The models have been ordered, from left to right, from the lowest to the highest value of $M_{ov}^{cor}$. (**b**) Lags *q*.

The time series *i* was always chosen as $\Psi _M^{cor}$ (top panel time series in Fig. S2) and *j* was varied between the temperature anomaly time series in the three subregions (the other three time series in Fig. S2). Figure 5 shows the results of *Q* and *q*, calculated for each one of the 10 CMIP5 models under consideration. *Q* varies substantially between the different models, with a maximum value of 0.72 for the temperature anomalies of the Subpolar Gyre in the model MRI-CGCM3, and a minimum of zero. Overall, the temperatures are considerably affected by the AMOC. The lags calculated with the index *q* are in almost all the cases negative, i.e. the temperature changes lag the AMOC changes. Furthermore, for the Subpolar Gyre, models with lower values of $M_{ov}^{cor}$, which supposedly have a less stable AMOC, show higher event synchronisation between extremes in the AMOC and temperature.

## Summary and discussion

In this paper, we analysed noise-induced transitions of the AMOC in an ensemble of CMIP5 models, and investigated the consequences of such transitions for the surface temperatures of the North Atlantic region. In each of the CMIP5 models, a considerable number of extreme AMOC events occur, events that cannot be attributed to the wind-stress variability, as measured through the Ekman transport. Transition probabilities were calculated through a threshold measure of $2 \sigma$ around the mean.

Models simply show too many AMOC transitions, then what is expected based on their $M_{ov}$ value; therefore, we proposed a correction to $M_{ov}$. This new indicator, $M_{ov}^{cor}$, was calibrated with the transition probabilities as obtained from a box model of the AMOC^23^. Although this was a bold step, internal consistency was demonstrated by the small spread in the integrated surface freshwater flux $F_S^{ext}$ which would arise under the absence of an AMOC. The average value obtained for $F_S^{ext}$ from the 10 CMIP5 models was $0.20 \pm 0.06$ Sv. The values of $M_{ov}^{cor}$ do not dramatically change with another choice (e.g. $3 \sigma$) of threshold for the AMOC transitions. The transition probabilities are sensitive to changes in $M_{ov}$: therefore, small errors in the estimation of the probabilities (still in the same order of magnitude) do not result in large errors in the estimation of $M_{ov}$.

The implication of this view is that the AMOC substantially influences the Atlantic freshwater flux $F_S$ and that all CMIP5 models considered are effectively in the multiple equilibrium regime. This could be investigated by applying large temporary localized freshwater fluxes and the results here would imply that collapsed AMOC states can be found. It would also be interesting, but certainly out of the scope of this paper, to compute $F_S^{ext}$ using a coupled ocean-atmosphere model. A slab-ocean version cannot be used as in these models a so-called *Q*-flux is prescribed, which already captures the effect of the AMOC on the freshwater flux^39^. One needs to set the atmospheric buoyancy fluxes which are transferred to the ocean to zero in a fully coupled simulation. The resulting equilibrium state may be far from any historical climate state but, if so, that would precisely demonstrate the impact of the presence of the AMOC on the present-day climate state.

Using event synchronisation, we studied connections between AMOC extreme events and temperatures over three North Atlantic regions in the CMIP5 models. The strength of the synchronisation substantially varies between the different models. Such differences were already found in another multimodel study, with a focus on correlations between decadal means of SST and the AMOC^28^. As expected, the synchronisation between AMOC and temperatures seems to occur for negative time lags, which means that the extreme events in the AMOC precede the ones in the temperatures. There is no robust connection between the stability of the AMOC, as measured by $M_{ov}^{cor}$, and the influence of the AMOC on the surface temperatures. Indeed, many other factors, such as a different background climate state, may affect this temperature response.

## Models and methods

In this work, pre-industrial control simulations from 10 different CMIP5 models were analysed and compared (see Table 1). Due to the availability of the specific data needed, we were not able to use other CMIP5 models. The choice of using control simulations is motivated by the fact that we are interested in noise-induced transitions of the AMOC, with the background climate state being in equilibrium.

The AMOC strength is represented by the maximum value (over depth) of the meridional overturning streamfunction at $26.5^\circ \hbox {N}$, from now indicated by $\Psi _M$. Midlatitude AMOC variations are partly induced by wind-stress variations, mainly through the Ekman transport^40^, which is a relatively fast response as it is accomplished by (barotropic) waves. The noise-induced transitions of the AMOC we are interested in are induced by high-frequency variations in the freshwater forcing^23^. Because of the nonlinear nature of the ocean circulation, it is generally not possible to separate both AMOC responses to buoyancy and wind-stress variations. Nevertheless, *Biastoch et al.*^41^ argue that such a separation is meaningful for midlatitude AMOC variability. Hence, we modify the AMOC time series by subtracting the Ekman transport at $26.5^\circ \hbox {N}$, indicated by $\Psi _E$ and expect that variations in the resulting time series $\Psi _M^{cor} = \Psi _M - \Psi _E$ better reflect those occurring through surface buoyancy fluctuations. Our choice is in agreement with the results shown by *Polo et al.*^42^, who conclude that buoyancy forcing dominates in the geostrophic transport anomalies $\Psi _M^{cor}$ (i.e., AMOC - Ekman transport) on decadal time scales, whereas wind-stress induced fluctuations are mostly confined to intraseasonal and interannual time scales.

To calculate the high-frequency variability of the freshwater flux over the Atlantic Ocean, i.e. the main component of the ‘noise’, we follow the same approach as in *Castellana et al.*^23^. First the integrated freshwater flux over the northern Atlantic region ($50^\circ \hbox {N}$–$70^\circ \hbox {N}$), indicated by $F^N_S$, and a southern region (south of $40^\circ \hbox {S}$), indicate by $F^S_S$, is calculated. Next, the difference $F^{NS}_S = F^N_S - F^S_S$ is determined and the ‘noise’ used here is the yearly-averaged component of $F^{NS}_S$, say $\bar{F}^{NS}_S$. The quantity $\eta$ below is the ratio between the standard deviation and the mean value of $\bar{F}^{NS}_S$, and it can be easily determined for each CMIP5 model.

To meaningfully define an F-type transition for a CMIP5 model time series, we recall the results for the box model of *Castellana et al.*^23^. Here, the strength of the AMOC was represented as the deep water flow in the North Atlantic (or downwelling). The F-type AMOC transitions were the consequence of a temporary cessation of the downwelling, without any subsequent reversal of the circulation. In a more realistic model, one can identify these transitions from the occurrence of extreme events in the AMOC time series, defined as values extending below a certain threshold. *Drijfhout et al.*^43^ used a similar approach to detect abrupt climate changes in CMIP5 models.

In the results below, the threshold used is $2\sigma$, where $\sigma$ is standard deviation of the $\Psi _M^{cor}$ time series. This choice seems to be a good compromise between two conditions that need to hold: on one hand, the threshold needs to be far enough from the mean, to guarantee that the occurrence of extreme events is well represented by a homogeneous Poisson process if the transitions are rare enough (formally when the threshold goes to infinity). On the other hand, enough values need to extend below the threshold, so that the analysis is statistically robust. Another precaution that needs to be taken is the fact that the events must be independent from each other: for this purpose, we clustered the events, using a 10-year window. Transition rates ($\lambda$) are computed, for each time series, by counting the number of extreme events per unit of time. The transition probabilities over a time interval *T* are then obtained using the following formula^44^

$$\begin{aligned} P(T) = 1- e^{-\lambda T} \end{aligned}$$

where, in most cases, we take $T = 100$ years.

Values of $M_{ov}$ of the CMIP5 models were provided by *Mecking et al.*^17^, who calculated $M_{ov}$ at $34^\circ \hbox {S}$ from historical simulations (in the period 1960–1989). Since the strength of the AMOC did not vary considerably during such time period, we assume that the corresponding values of $M_{ov}$ from control simulations do not differ much from the values in *Mecking et al.*^17^. This was confirmed for two of the models: we calculated $M_{ov}$ for pre-industrial control simulations from MIROC5 and MRI-CGCM3. In both cases, the absolute error in the estimation of $M_{ov}$ is about 0.02 Sv. Table 1 contains the $M_{ov}$ values of the models considered: four of them are positive, which would indicate^14^ that the AMOC is in a single equilibrium regime and hence no noise-induced transitions are expected.

## Supplementary information

Supplementary material 1

## References

[1] Smeed, D. *et al.* Atlantic Meridional Overturning Circulation observed by the RAPID-MOCHA-WBTS (RAPID-Meridional Overturning Circulation and heatflux array-western boundary time series) array at $$26^\circ \text{N}$$ from 2004 to 2018. British Oceanographic Data Centre - Natural Environment Research Council, UK. 10.5285/8cd7e7bb-9a20-05d8-e053-6c86abc012c2 (2019).

[2] Stommel H (1961). Thermohaline convection with two stable regimes of flow. Tellus.

[3] Bryan F (1986). High-latitude salinity effects and interhemispheric thermohaline circulations. Nature.

[4] Cheng W, Chiang JC, Zhang D (2013). Atlantic Meridional Overturning Circulation (AMOC) in CMIP5 models: RCP and historical simulations. J. Clim..

[5] Cessi P, Young W (1992). Multiple equilibria in two-dimensional thermohaline circulation. J. Fluid Mech..

[6] Weijer W, Ruijter WPMD, Dijkstra HA (2001). Stability of the Atlantic Overturning Circulation: competition between Bering Strait freshwater flux and Agulhas heat and salt sources. J. Phys. Oceanogr..

[7] Rahmstorf S (2005). Thermohaline circulation hysteresis: a model intercomparison. Geophys. Res. Lett..

[8] Hawkins E (2011). Bistability of the Atlantic Overturning Circulation in a global climate model and links to ocean freshwater transport. Geophys. Res. Lett..

[9] Smeed D (2018). The North Atlantic Ocean is in a state of reduced overturning. Geophys. Res. Lett..

[10] Taylor KE, Stouffer RJ, Meehl GA (2012). An overview of CMIP5 and the experiment design. Bull. Am. Meteorol. Soc..

[11] Gent PR (2018). A commentary on the Atlantic Meridional Overturning Circulation stability in climate models. Ocean Model..

[12] Drijfhout SS, Weber SL, van der Swaluw E (2011). The stability of the MOC as diagnosed from model projections for pre-industrial, present and future climates. Clim. Dyn..

[13] Weijer W (2019). Stability of the Atlantic Meridional Overturning Circulation: a review and synthesis. J. Geophys. Res.: Oceans.

[14] de Vries P, Weber SL (2005). The Atlantic freshwater budget as a diagnostic for the existence of a stable shut down of the Meridional Overturning Circulation. Geophys. Res. Lett..

[15] Huisman SE, den Toom M, Dijkstra HA, Drijfhout S (2010). An indicator of the multiple equilibria regime of the Atlantic Meridional Overturning Circulation. J. Phys. Oceanogr..

[16] Cimatoribus AA, Drijfhout SS, Dijkstra HA (2012). Meridional Overturning Circulation: stability and ocean feedbacks in a box model. Clim. Dyn..

[17] Mecking JV, Drijfhout SS, Jackson LC, Andrews MB (2017). The effect of model bias on Atlantic freshwater transport and implications for AMOC bi-stability. Tellus A: Dyn. Meteorol. Oceanogr..

[18] Weaver AJ (2012). Stability of the Atlantic Meridional Overturning Circulation: a model intercomparison. Geophys. Res. Lett..

[19] Deshayes J (2013). Oceanic hindcast simulations at high resolution suggest that the Atlantic MOC is bistable. Geophys. Res. Lett..

[20] Liu W, Liu Z, Brady EC (2014). Why is the AMOC monostable in coupled General Circulation Models?. J. Clim..

[21] Bryden HL, King BA, McCarthy GD (2011). South Atlantic Overturning Circulation at $$24^\circ \text{ S }$$. J. Mar. Res..

[22] Ashwin P, Wieczorek S, Vitolo R, Cox P (2012). Tipping points in open systems: bifurcation, noise-induced and rate-dependent examples in the climate system. Philos. Trans. R. Soc. A: Math. Phys. Eng. Sci..

[23] Castellana D, Baars S, Wubs F, Dijkstra H (2019). Transition probabilities of noise-induced transitions of the Atlantic Ocean Circulation. Sci. Rep..

[24] Latif M (2004). Reconstructing, monitoring, and predicting multidecadal-scale changes in the North Atlantic thermohaline circulation with sea surface temperature. J. Clim..

[25] Kloewer M, Latif M, Ding H, Greatbatch RJ, Park W (2014). Atlantic Meridional Overturning Circulation and the prediction of North Atlantic sea surface temperature. Earth Planet. Sci. Lett..

[26] Palter JB (2015). The role of the Gulf Stream in European climate. Ann. Rev. Mar. Sci..

[27] Drijfhout S (2015). Competition between global warming and an abrupt collapse of the AMOC in Earth’s energy imbalance. Sci. Rep..

[28] Roberts CD, Garry FK, Jackson LC (2013). A multimodel study of sea surface temperature and subsurface density fingerprints of the Atlantic Meridional Overturning Circulation. J. Clim..

[29] Rahmstorf S (2015). Exceptional twentieth-century slowdown in Atlantic Ocean Overturning Circulation. Nat. Clim. Change.

[30] Duchez A (2016). Potential for seasonal prediction of Atlantic sea surface temperatures using the RAPID array at $$26^\circ \text{ N }$$. Clim. Dyn..

[31] Von Storch H, Zwiers FW (2001). Statistical Analysis in Climate Research.

[32] Toom MD, Dijkstra HA, Cimatoribus AA, Drijfhout SS (2012). Effect of atmospheric feedbacks on the stability of the Atlantic Meridional Overturning Circulation. J. Clim..

[33] Liu W, Xie S-P, Liu Z, Zhu J (2017). Overlooked possibility of a collapsed Atlantic Meridional Overturning Circulation in warming climate. Sci. Adv..

[34] Wood RA, Rodríguez JM, Smith RS, Jackson LC, Hawkins E (2019). Observable, low-order dynamical controls on thresholds of the Atlantic Meridional Overturning Circulation. Clim. Dyn..

[35] Cimatoribus AA, Drijfhout SS, Den Toom M, Dijkstra HA (2012). Sensitivity of the Atlantic Meridional Overturning Circulation to South Atlantic freshwater anomalies. Clim. Dyn..

[36] Quiroga RQ, Kreuz T, Grassberger P (2002). Event synchronization: a simple and fast method to measure synchronicity and time delay patterns. Phys. Rev. E.

[37] Malik N, Marwan N, Kurths J (2010). Spatial structures and directionalities in Monsoonal precipitation over South Asia. Nonlinear Process. Geophys..

[38] Stolbova V, Tupikina L, Bookhagen B, Marwan N, Kurths J (2014). Topology and seasonal evolution of the network of extreme precipitation over the Indian subcontinent and Sri Lanka. Nonlinear Process. Geophys..

[39] L’hévéder B, Codron F, Ghil M (2015). Impact of anomalous northward oceanic heat transport on global climate in a slab ocean setting. J. Clim..

[40] Zhao J, Johns W (2014). Wind-forced interannual variability of the Atlantic Meridional Overturning Circulation at $$26.5^\circ \text{ N }$$. J. Geophys. Res.: Oceans.

[41] Biastoch A, Böning C, Getzlaff J, Molines J, Madec G (2008). Causes of interannual-decadal variability in the Meridional Overturning Circulation of the midlatitude North Atlantic Ocean. J. Clim..

[42] Polo I, Robson J, Sutton R, Balmaseda MA (2014). The importance of wind and buoyancy forcing for the boundary density variations and the geostrophic component of the AMOC at $$26^\circ \text{ N }$$. J. Phys. Oceanogr..

[43] Drijfhout S (2015). Catalogue of abrupt shifts in Intergovernmental Panel on Climate Change climate models. Proc. Natl. Acad. Sci..

[44] Coles S, Bawa J, Trenner L, Dorazio P (2001). An Introduction to Statistical Modeling of Extreme Values.

