# Supplementary material for: Noise-induced transitions of the Atlantic Meridional Overturning Circulation in CMIP5 models
Source: Sci Rep. 2020 Nov 18;10:20040. doi: 10.1038/s41598-020-76930-5 (PMC7674444; doi:10.1038/s41598-020-76930-5)
Supplement: Supplementary file 1 — Supplementary material 1 [file 41598_2020_76930_MOESM1_ESM.pdf]

Supporting Information for
"Noise-induced Transitions of the Atlantic Meridional Overturning Circulation in CMIP5 models"

Daniele Castellana[1,*] **and Henk A. Dijkstra**[1,2]

[1]Institute for Marine and Atmospheric research Utrecht, Department of Physics, Utrecht University, Utrecht, The Netherlands.
[2]Centre for Complex Systems Studies, Department of Physics, Utrecht University, Utrecht, The Netherlands.
[*]d.castellana@uu.nl

Contents

1. Section A: Temperatures in the North Atlantic
2. Section B: Event synchronisation algorithm

Section A: Temperatures in the North Atlantic

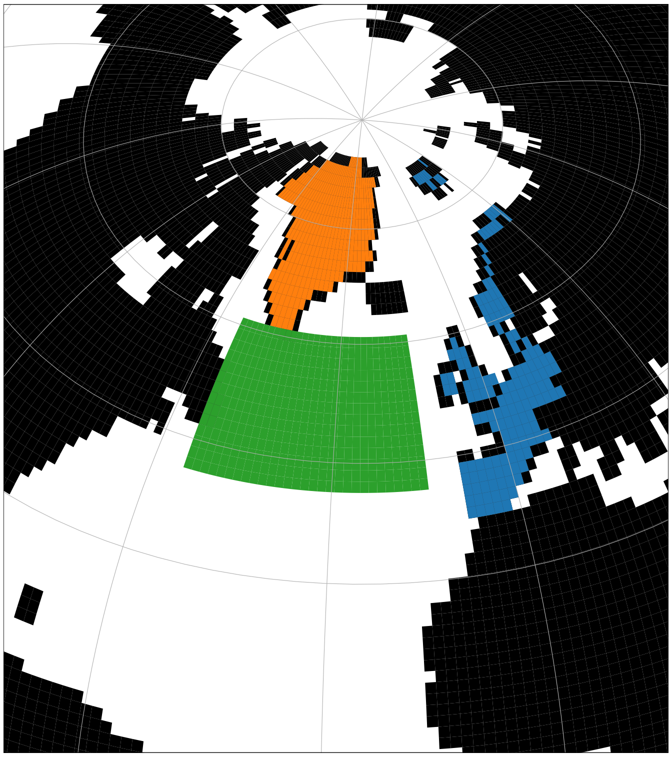

Figure S1. Masks corresponding to three subregions of the North Atlantic: Western Europe, Greenland and Subpolar Gyre ([40°N, 60°N] × [55°W, 15°W]). The map was created using the Python library Cartopy (version 0.17.0)[1].

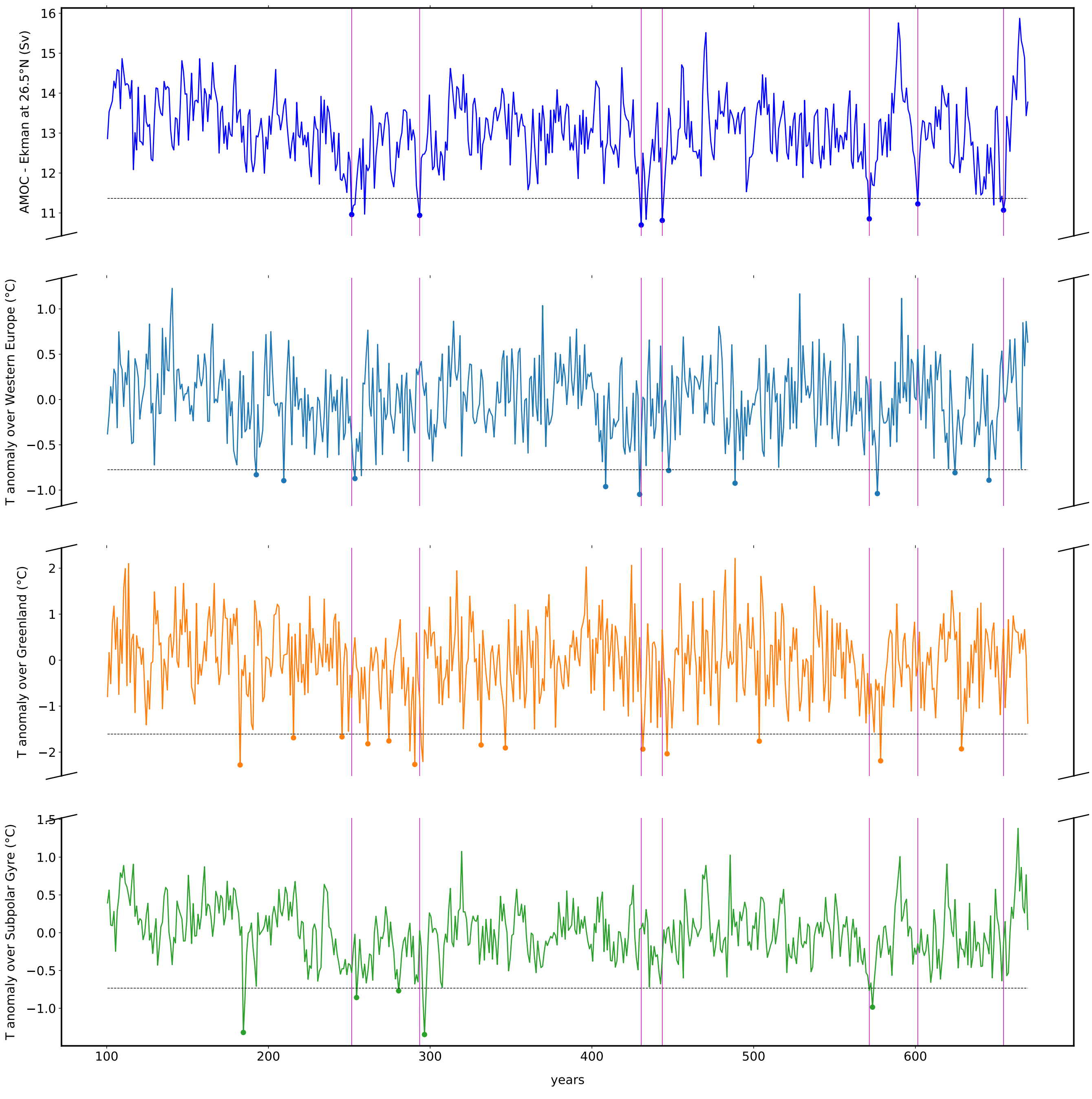

Figure S2. *The upper panel shows the yearly-averaged time series of* Ψ_M^{cor}*, i.e. the buoyancy-driven component of the AMOC, in the pre-industrial control simulation of the model MIROC5. The dashed horizontal line indicates the lower bound of the* 2σ *confidence interval around the mean. The circles identify the time points at which extreme events occur, defined as the values extending below the confidence interval. Note that some of the values were not marked, because of the clustering. The other three panels are the SST anomalies calculated over the three subregions indicated in Fig. S1. The magenta vertical lines are drawn in correspondence with the extreme events of* Ψ_M^{cor} *on top of all the time series. In this way, it is possible to qualitatively check whether a certain extreme event in the AMOC is related with anomalies in the surface temperatures.*

Section B: Event synchronisation algorithm

The Event Synchronisation (ES) algorithm, conceived by *Quiroga et al.*[2] and improved by *Malik et al.*[3], works as follows: consider two time series, labelled with indices i and j, and a set of events occurring at time t_l^i, with $l = 1,2,\dots,s_i$ for the time series i and at time t_m^j, with $m = 1,2,\dots,s_j$ for the time series j, respectively. Two events l and m are considered to be synchronised within a time lag $\pm\tau_{lm}^{ij}$ if $0 < t_l^i - t_m^j < \tau_{lm}^{ij}$, where $\tau_{lm}^{ij} = \min\{t_{l+1}^i - t_l^i, t_l^i - t_{l-1}^i, t_{m+1}^j - t_m^j, t_m^j - t_{m-1}^j\}/2$. The synchronisation index for the two events is defined as:

$$J_{lm}^{ij} = \begin{cases} 1 & \text{if } 0 < t_l^i - t_m^j < \tau_{lm}^{ij}, \\ 1/2 & \text{if } t_l^i = t_m^j, \\ 0 & \text{otherwise} \end{cases} \tag{S1}$$

Now it is possible to count the number of times two events occurring in the two time series are synchronised (with the event in i preceding the one in j):

$$c^{ij} = \sum_{l=1}^{s_i} \sum_{m=1}^{s_j} J_{lm}^{ij} \tag{S2}$$

and vice versa c^{ji}. The strength of the synchronisation is defined as

$$Q^{ij} = \frac{c^{ij} + c^{ji}}{\sqrt{(s_i - 2)(s_j - 2)}} \tag{S3}$$

and normalised, such that $0 \leq Q^{ij} \leq 1$, for each pair of time series i and j. $Q^{ij} = 1$ means full synchronisation (i.e. between a time series with itself), while $Q^{ij} = 0$ indicates absence of synchronisation. With the algorithm, it is also possible to check the delay between the events: this is measured by calculating another index

$$q^{ij} = \frac{c^{ij} - c^{ji}}{\sqrt{(s_i - 2)(s_j - 2)}} \tag{S4}$$

When q^{ij} is positive (negative), the events in the time series j precede (follow) the events in the time series i.

References

1. Met Office. *Cartopy: a cartographic python library with a matplotlib interface*. Exeter, Devon (2010 - 2015).
2. Quiroga, R. Q., Kreuz, T. & Grassberger, P. Event synchronization: a simple and fast method to measure synchronicity and time delay patterns. *Phys. review E* **66**, 041904, DOI: https://doi.org/10.1103/PhysRevE.66.041904 (2002).
3. Malik, N., Marwan, N. & Kurths, J. Spatial structures and directionalities in Monsoonal precipitation over South Asia. *Nonlinear Process. Geophys.* **17**, 371–381, DOI: https://doi.org/10.5194/npg-17-371-2010 (2010).
